# The Phenolics from the Roots of *Livistona chinensis* Show Antioxidative and Obsteoblast Differentiation Promoting Activity

**DOI:** 10.3390/molecules19010263

**Published:** 2013-12-27

**Authors:** Xiaobin Zeng, Jun Tian, Liao Cui, Yang Wang, Yanjie Su, Xin Zhou, Xiangjiu He

**Affiliations:** 1 Guangdong Key Laboratory for Research and Development of Natural Drugs, Department of Pharmacology, Guangdong Medical College, Zhanjiang 524023, Guangdong, China; E-Mails: cuiliao@163.com (L.C.); super126su@126.com (Y.S.); 18820648019@163.com (X.Z.); 2 College of Life Science, Jiangsu Normal University, Xuzhou 221116, Jiangsu, China; E-Mail: tj-085@163.com; 3 Shenzhen Xinpeng Shengwu Gongcheng Co. LTD, Shenzhen 518055, Guangdong, China; E-Mail: cherryyaya@fomail.com; 4 School of Pharmaceutical Sciences, Wuhan University, Wuhan 430071, Hubei, China

**Keywords:** phenolic, flavane, *Livistona chinensis*, osteoporosis, oxidative stress

## Abstract

This study investigated the antioxidative and obsteoblast differentiation promoting activity of the phenolics isolated from the 70% ethanol extract of the roots of *Livistona chinensis*. Two new phenolics, (2*R*,3*R*)-3,5,6,7,3',4'-hexahydroxyflavane (**1**), and phenanthrene-2,4,9-triol (**2**), together with six known phenolics **3**–**8**, were isolated and identified on the basis of extensive spectroscopic analysis. The antioxidative and obsteoblast differentiation promoting abilities of the compounds **1**–**3**, **7**–**8** were tested, the phenolics **1**–**3**, **7** showed effects on proliferation of osteoblastic cells and antioxidative activity of 3.125–50 µg/mL. In addition, the phenolics **1**–**3** observably increased alkaline phosphatase activity, osteocalcin content and hydroxyproline content in osteoblastic cells. Phenolic **1** at 12.5 µg/mL concentration significantly increased the area of nodules by about 9.35-fold. The antioxidative activity results indicated that the anti-osteoporosis effects of these phenolics may be linked to a reduction of oxidative stress. The observed effects of these phenolics on bone formation by rat osteoblastic cells suggest that these phenolics may have beneficial effects on bone health.

## 1. Introduction

Osteoporosis is a disease characterized by the loss of bone mass and degeneration of bone microstructure, resulting in an increased risk of fractures. Osteoporosis, called postmenopausal osteoporosis, is common in women after menopause [1]. It may also develop in men, especially in the aged man, which is called age-related bone loss [2]. Osteoporosis may significantly affect life expectancy and quality of life in humans.

Oxidative stress, resulting from excessive formation of reactive oxygen species (ROS) or dysfunction of antioxidant defense system, represents a major cause of age-associated pathological conditions including aging [3] and postmenopausal bone loss [4]. Oxidative stress is a pivotal pathogenic factor for age-related bone loss in mice, leading to an increase in osteoblast and osteocyte apoptosis and a decrease in osteoblast number and the rate of bone formation [5,6]. On the other hand, ROS is also involved in bone resorption with a direct contribution of osteoclast-generated superoxide to bone degradation [7,8]. Oxidative stress increases differentiation and function of osteoclasts [8].

**Figure 1:**
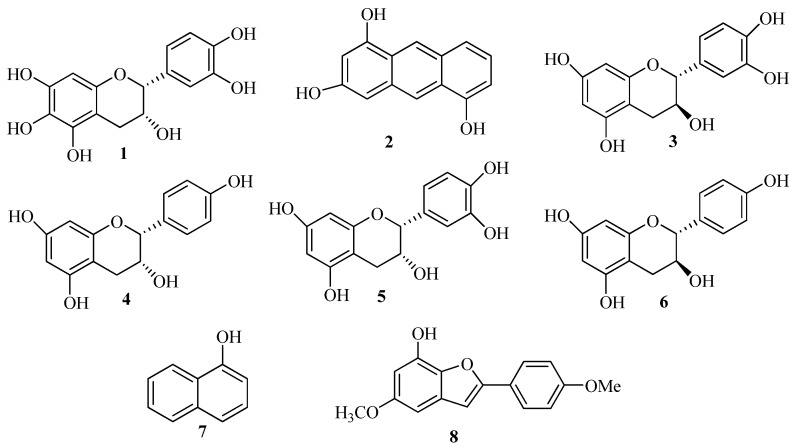
Chemical structures of the phenolics **1**–**8**.

The genus *Livistona* is widely distributed in several ecosystems throughout the tropical zone, including upland hardwoods, flatwoods, and tropical hammocks. *L. chinensis* is commonly used for analgesic and hemostatic purposes [9,10]. Phytochemically, it has been reported to contain flavonoids, phenolics, ceramides and glycerides [11,12,13,14,15,16,17]. As part of our ongoing search for active anti-osteoporosis compounds from traditional medicinal plants, we investigated the constituents from the roots of *L. chinensis*. Eight phenolics, including two new phenolics, (2*R*,3*R*)-3,5,6,7,3',4'-hexahydroxyflavane (**1**), phenanthrene-2,4,9-triol (**2**), and six known compounds **3**–**8** was isolated and identified from the ethyl acetate-soluble fraction of a 70% ethanol extract. The structures of phenolics **1**–**8** are shown in Figure 1. Their potential osteogenic effects on the proliferation, differentiation and mineralization of osteoblastic cells were evaluated. To clarify the underlying mechanisms of action of the phenolics, we investigated whether protection against osteoporosis was linked to a reduction of oxidative stress.

## 2. Results and Discussion

### 2.1. Structure Elucidation of the New Phenolics **1** and **2**

Phenolic **1** was obtained as an amorphous red powder. The positive electrospray ionization mass spectrometry (ESI-MS) gave a [M+Na]^+^ ion at *m/z* 329 from which in combination with its ^1^H and ^13^C-NMR data the molecular formula of C_15_H_14_O_7_ was inferred. The ^1^H-NMR spectrum of **1** exhibited three *meta*-coupled doublets at *δ*_H_ 6.94 (1H, d, *J* =1.6 Hz, H-2'), 6.72 (1H, d, *J* = 8.4 Hz, H-5') and 6.76 (1H, dd, *J* = 8.4, 2.0 Hz, H-6'), consistent with a flavane 1',3',4'-trisubstituted B ring. One *meta*-coupled doublet at *δ*_H_ 5.91 (1H, s, H-8) was consistent with a flavane 5,6,7-trioxygenated A ring. These resonances, together with *δ*_H_ 4.77 (1H, brs, H-2), 4.14 (1H, m, H-3), 2.83 (1H, dd, *J* = 16.4, 4.8 Hz, H-4ax) and 2.70 (1H, dd, *J* = 16.8, 2.8 Hz, H-4eq) and the corresponding carbon signals in the HMQC spectrum revealed that **1** was 3,5,6,7,3',4'-hexahydroxyflavane. The following spectroscopic analysis made the absolute configurations at C-2 and C-3 assignable. The small coupling constants of H-2 and H-3 were indicative of the 2,3-*cis* relative configuration with the 2-phenyl group in a pseudo-equatorial orientation and the 3-hydroxy group in a pseudo-axial orientation, that’s to say, the configurations could be (2*S*,3*S*) or (2*R*,3*R*) [14]. By comparison with the literature values of 2*R*,3*R*-3,5,6,7,8,4'-hexahydroxyflavane, whose optical rotation value {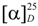 = −48.0 (c 0.30, MeOH)} indicated a 2*R* and 3*R* absolute configuration [14], the optical rotation value of **1** {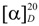 = −43.0 (c0.3, MeOH)} suggested the absolute configurations of C-2 and C-3 were (2*R*, 3*R*). Thus, the structure of **1** was elucidated to be 2*R*,3*R*-3,5,6,7,3',4'-hexahydroxyflavane.

Phenolic **2** was obtained as an amorphous powder. Positive electrospray ionization mass spectrometry (ESI-MS) produced a [M+Na]^+^ ion at *m/z* 249, indicating a mass of 226, which is compatible with the molecular formula C_14_H_10_O_3_. Its molecular formula was confirmed by HR-ESIMS, which showed the ion [M+Na]^+^ at *m/z* 249.0637 (calcd. 249.0630). In the ^1^H-NMR spectrum, there are seven signals at δ_H_ 6.15 (1H, t, *J* = 5.6 Hz, H-11), 6.44 (1H, d, *J* = 2.0 Hz, H-14), 6.76 (1H, d, *J* = 8.8 Hz, H-10), 6.81 (1H, s, H-3), 6.92 (1H, s, H-5), 6.97 (1H, s, H-7) and 7.34 (1H, d, *J* = 8.4 Hz, H-12). The ^13^C-NMR spectrum included 14 nonequivalent carbon signals at δ_C_ 102.9–159.8 ppm, implying phenolic **2** is an anthracene compound. The counpling constant of every hydrogen mentioned above indicates the assignment of the hydroxyl groups at C-2, C-4 and C-9. From the data above, phenolic **2** was identified as a new compound, anthracene-2,4,9-triol. The known phenolics were identified, by comparing of their spectroscopic data with data previously reported in the literature, as (−)-catechin (**3**) [14], (−)-epiafzelechin (**4**) [14], (−)-epicatechin (**5**) [18], (−)-afzelechin (**6**) [18], naphthalen-2-ol (**7**) [19] and 7-hydroxy-5,4'-dimethoxy-2-arylbenzofuran (**8**) [15].

### 2.2. *In Vitro* Effect of the Phenolics **1**–**3**, **7**, **8** in Osteoblast Viability

When different concentration of the phenolics **1**–**3**, **7**–**8** were cultured with rat osteoblast cells. Phenolic **8** inhibited the rat osteoblast cell growth and proliferation. However, phenolics **1** and **3** at concentration from 3.125 µg/mL to 50 µg/mL stimulated rat osteoblast cell growth and proliferation, and this effect appeared in a dose-dependent manner (Table 1). We observed a significant increase in promoting proliferative activity in cells treated with phenolic **1** after 2 days of treatment. Phenolic **1** at 25 µg/mL concentration showed the highest promoting proliferative activity (about 2.14-fold) at the second day after treatment, when compared with the negative control, which exhibited more potential promoting activity than positive control-resveratrol.

**Table 1:** Effects of the phenolics **1**–**3**, **7** on the proliferation of rat osteoblast cells after 48 h, as determined by MTT assay.

Concentrations (μg/mL)	Cell Viability (%)				
	1	2	3	7	Positive Control
0	101.73 ± 6.54	104.55 ± 8.78	103.29 ± 10.85	101.29 ± 9.85	101.34 ± 13.23
3.125	158.43 ± 15.44 *	111.75 ± 12.19 *	163.99 ± 10.50 *	107.99 ± 10.50	107.21 ± 9.56
6.25	181.54 ± 8.19 *	125.07 ± 10.23 *	171.45 ± 15.44 *	111.45 ± 11.44 *	115.25 ± 14.14 *
12.5	208.88 ± 12.95 *	129.13 ± 8.19 *	186.90 ± 16.78 *	116.90 ± 9.78 *	169.32 ± 16.00 *
25	314.67 ± 21.48 *	134.23 ± 11.97 *	214.22 ± 20.60 *	121.22 ± 12.60 *	197.66 ± 16.57 *
50	228.94 ± 11.58 *	125.76 ± 11.60 *	175.73 ± 16.59 *	115.73 ± 8.59 *	153.23 ± 16.65 *

Note: * *p* ˂ 0.05 *vs*. negative control.

### 2.3. Effects of the Phenolics **1**–**3**, **7** on ALP Activity of Rat Osteoblast

**Figure 2:**
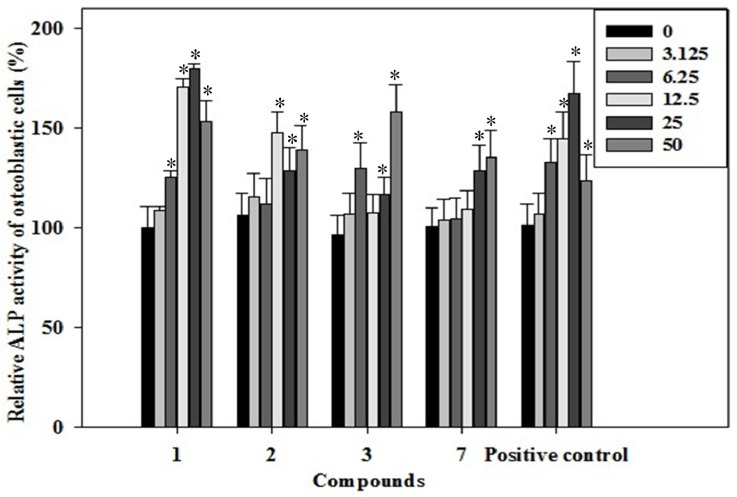
The effects of the phenolics (**1**–**3**, **7**) on ALP activity were assessed in rat osteoblast cells treated with the phenolics (**1**–**3**, **7**) (3.125–50 µg/mL) for 7 days.

Note: * *p* ˂ 0.05 *vs.* negative control.

The phenotype of mature osteoblasts is characterized by their ability to synthesize and secrete molecules of the extracellular matrix. Osteoblasts regulate their mineralization of the formed matrix by producing alkaline phosphatase (ALP) [20]. This enzyme hydrolyzes phosphate esters to increase the local phosphate concentration and enhance mineralization of the extracellular matrix [21]. One of the characteristics of a mature osteoblast phenotype is the ability of the cells to synthesize ALP, which is considered an early marker of osteoblast differentiation. ALP is an early marker of osteogenic differentiation [22]. To determine whether the phenolics **1**–**3**, **7** could stimulate osteogenic differentiation, the effects of the new flavane on bone formation early marker, ALP activity, was measured. Our data illustrated that treatment of rat osteoblast cells with the phenolics **1**–**3**, **7** stimulated ALP activity in a dose-dependent manner. As shown in Figure 2, phenolic **1** at most of the treated concentrations increased the ALP activity compared with the negative control. At 7 day after treatment, phenolic **1** showed a dose-dependent increase in ALP activity by 8.8%, 25.8%, 70.4%, 80.1% and 53.3% at the concentrations of 3.125, 6.25, 12.5, 25 and 50 μg/mL, respectively, as compared to the control. Phenolic **2** demonstrated a dose-dependent increase in ALP activity by 11.5%, 11.7%, 48.0%, 28.8% and 39.3% at the concentrations of 3.125, 6.25, 12.5, 25 and 50 μg/mL, respectively, as compared to the control. Phenolic **3** significantly increased the ALP activity by 6.9%, 29.7%, 7.4%, 16.8% and 58.1% at concentrations of 3.125, 6.25, 12.5, 25 and 50 μg/mL, as compared to the control. Phenolic **7** significantly increased the ALP activity by 6.9%, 4.7%, 9.4%, 28.8% and 35.1% at concentrations of 3.125, 6.25, 12.5, 25 and 50 μg/mL, as compared to the control. At 25 μg/mL, the ALP activity of the new flavane was significantly increased by about 0.80-fold when compared with the negative control at 7 day after treatment. Its osteogenic effect was stronger than the positive control resveratrol at 25 μg/mL.

### 2.4. Effects of the Phenolics **1**–**3**, **7** on Osteocalcin Content of the Rat Osteoblastic Cells

**Figure 3:**
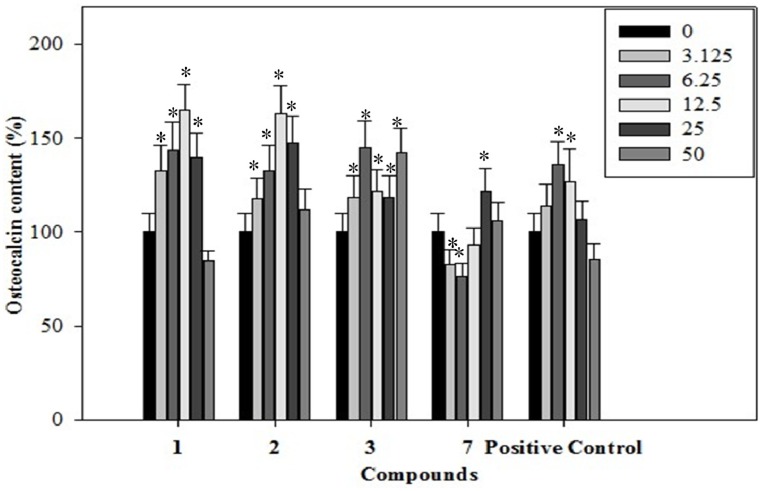
The effects of the phenolics (**1**–**3**, **7**) on osteocalcin content were assessed in rat osteoblast cells treated with the phenolics (**1**–**3**, **7**) (3.125–50 µg/mL) for 14 days.

Note: * *p* ˂ 0.05 *vs.* negative control.

Osteocalcin is a noncollagenous protein found in bone and dentin. Osteocalcin is secreted solely by osteoblasts and thought to play a role in the body's metabolic regulation and is pro-osteoblastic, or bone-building, by nature. It is also implicated in bone mineralization and calcium ion homeostasis [23]. It is often used as a marker for the bone formation process. The effects of the phenolics **1**–**3**, **7** on the bone formation marker osteocalcin were determined. Our data illustrated that treatment of rat osteoblast cells with the phenolics **1**–**3**, **7** for 14 days stimulated osteocalcin secreting in a dose-dependent manner. As shown in Figure 3, phenolic **1** demonstrated a dose-dependent increase in osteocalcin content by 32.6%, 43.9%, 65.3% and 39.6% at the concentrations of 3.125, 6.25, 12.5 and 25 μg/mL, as compared to the control. Phenolic **2** demonstrated a dose-dependent increase in osteocalcin content by 18.0%, 32.8%, 63.1%, 47.6% and 11.7% at the concentrations of 3.125, 6.25, 12.5, 25 and 50 μg/mL, as compared to the control. Phenolic **3** demonstrated a dose-dependent increase in osteocalcin content by 18.3%, 45.0%, 21.5%, 18.4% and 42.1% at the concentrations of 3.125, 6.25, 12.5, 25 and 50 μg/mL, as compared to the control. Phenolic **7** demonstrated a dose-dependent increase in osteocalcin content by 21.6% and 5.8% at the concentrations of 25 and 50 μg/mL, as compared to the control. Treated with 12.5 μg/mL phenolic **1**, the osteocalcin content was significantly increased by about 0.65-fold when compared with the negative control. Its osteogenic effect was stronger than the positive control resveratrol at 6.25 μg/mL.

### 2.5. Effects of the Phenolics **1**–**3**, **7** on Collagen Content of the Rat Osteoblastic Cells

**Figure 4:**
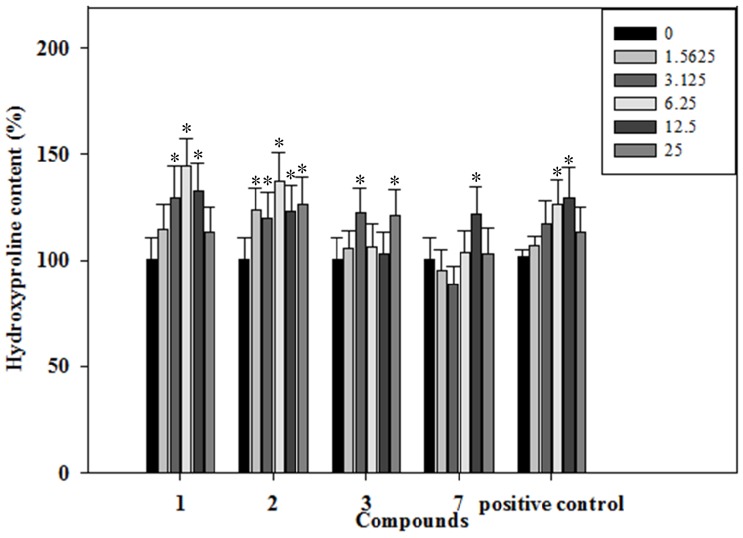
The effects of the phenolics (**1**–**3**, **7**) on the hydroxyproline content were determined in rat osteoblast cells treated with the phenolics (**1**–**3**, **7**) (1.5625–25 µg/mL) for 22 days.

Note: * *p* ˂ 0.05 *vs.* negative control.

Collagen type I is a marker of osteogenic maturity, and bone is a matrix with collagen type I [24]. Collagen content of the rat osteoblastic cells was determined by a hydroxyproline assay. The effects of the phenolics **1**–**3**, **7** on bone formation of the intermediate-term marker, collagen, were determined. Our data illustrated that treatment of rat osteoblast cells with the phenolics **1**–**3**, **7** for 22 days stimulated collagen secreting in a dose-dependent manner. As shown in Figure 4, phenolic **1** demonstrated a dose-dependent increase in collagen content by 14.3%, 29.5%, 44.4%, 32.7% and 13.2% at the concentrations of 1.5625, 3.125, 6.25, 12.5 and 25 μg/mL, as compared to the control. Phenolic **2** demonstrated a dose-dependent increase in collagen content by 23.3%, 19.5%, 37.5%, 22.7% and 26.3% at the concentrations of 1.5625, 3.125, 6.25, 12.5 and 25 μg/mL, as compared to the control. Phenolic **3** demonstrated a dose-dependent increase in collagen content by 5.2%, 22.4%, 6.4%, 2.7% and 21.2% at the concentrations of 1.5625, 3.125, 6.25, 12.5 and 25 μg/mL, as compared to the control. Phenolic **7** demonstrated a dose-dependent increase in collagen content by 3.4%, 21.6% and 3.2% at the concentrations of 6.25, 12.5 and 25 μg/mL, as compared to the control. Treated with 6.25 μg/mL phenolic **1**, the collagen content was significantly increased by about 0.44-fold when compared with the negative control. Its osteogenic effect was stronger than the positive control resveratrol at 50 μg/mL.

### 2.6. Effects of the Phenolics **1**–**3**, **7** on the Formation of Mineralized Bone Nodules

**Figure 5:**
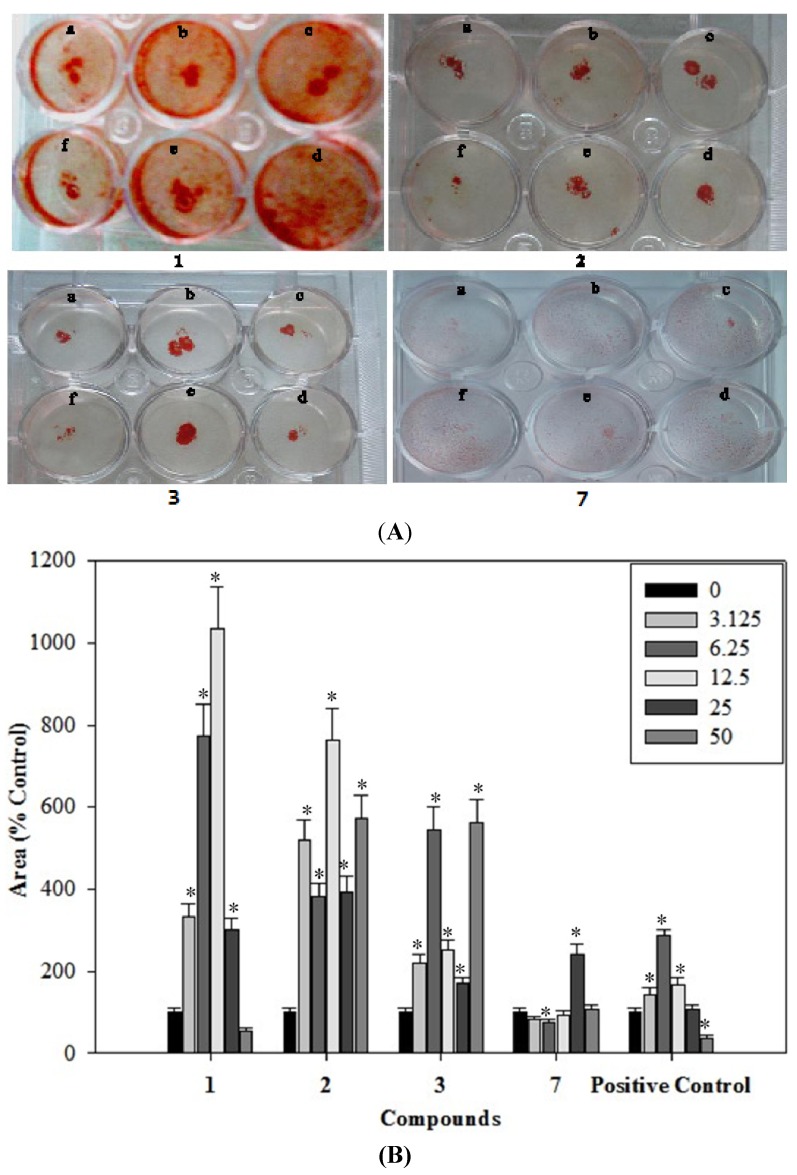
Dose-dependent effects of the phenolics **1**–**3**, **7** on the formation of mineralized nodules in a 12-well plate fixed on Day 20 and stained by alizarin red. (**A**) Cells were treated with the phenolics **1**–**3**, **7** at concentrations of 3.125, 6.25, 12.5, 25, 50 and 0 µg/mL (from a to f) on day 20; and (**B**) Dose-dependent effects of the phenolics **1**–**3**, **7** on the area of mineralized bone nodules stained by alizarin red.

Note: * *p* ˂ 0.05 *vs.* negative control.

The mineralized bone nodules formed by rat osteoblast cells were observed after 20 days of treatment. The mineralized bone nodules could be visualized by the naked eye as red-purple spots after staining with alizarin red (Figure 5A). The addition of phenolic **1** at concentrations as low as 3.125 µg/mL to the culture media increased the formation of mineralized nodules compared with control. Phenolics **1**–**3**, **7** caused a dose-dependent increase in the area of mineralized bone nodules as quantified using a Image-Pro Plus 6.0 software (Figure 5B). Phenolic **1** demonstrated a dose-dependent increase in the area of mineralized bone nodules by 232.6%, 673.9%, 935.3% and 199.6% at the concentrations of 3.125, 6.25, 12.5 and 25 μg/mL, as compared to the control. Phenolic **2** demonstrated a dose-dependent increase in the area of mineralized bone nodules by 518.0%, 282.8%, 663.1%, 290.6% and 571.7% at the concentrations of 3.125, 6.25, 12.5, 25 and 50 μg/mL, as compared to the control. Phenolic **3** demonstrated a dose-dependent increase in the area of mineralized bone nodules by 118.3%, 445.0%, 151.5%, 68.4% and 462.1% at the concentrations of 3.125, 6.25, 12.5, 25 and 50 μg/mL, as compared to the control. Phenolic **7** demonstrated a dose-dependent increase in the area of mineralized bone nodules by 141.6% and 5.8% at the concentrations of 25 and 50 μg/mL, as compared to the control. Analysis of the results from alizarin red staining showed that at 12.5 µg/mL, phenolic **1** increased the area of nodules by about 9.35-fold.

### 2.7. Antioxidantive Activity of the Phenolics **1**–**3**, **7**

#### 2.7.1. Protective Effects against H_2_O_2_-Induced Cell Injury in C2C12 Mouse Myoblast Cells

To investigate the protective effects of phenolics **1**–**3**, **7** on H_2_O_2_-induced cytotoxicity, C2C12 mouse myoblast cells were cultured in the presence of 100 μM H_2_O_2_ with or without treatment with phenolics **1**–**3**, **7**. Treatment with phenolics **1**–**3**, **7** increased the cell viability of C2C12 mouse myoblast cells compared to the H_2_O_2_-treated control (Table 2A). Treatment with phenolics **1**–**3**, **7** decreased the number of apoptotic cells compared to the H_2_O_2_-treated control group (Table 2B). From the results, phenolics **1**–**3**, **7** isolated from the roots of *L. chinensis* showed potential protective effects against H_2_O_2_-induced cell injury in C2C12 mouse myoblast cells. Flavane **1** at 50 µg/mL concentration showed the highest protective activity.

**Table 2:** Protective effects of the phenolics **1**–**3**, **7** on H_2_O_2_-induced cell injury in rat osteoblastic cells. Rat osteoblast cells were cultured in the presence of 100 μM H_2_O_2_ with or without treatment of the phenolics **1**–**3**, **7** different concentrations. (**A**) The cell viability, and (**B**) the cell apoptosis, were assessed by MTT assay and flow cytometric analysis, respectively.

**Concentrations (μg/mL)**	**(A) Cell Viability (%)**				
	**1**	**2**	**3**	**7**	**Positive Control**
**0**	211.23 ± 19.57 *	205.92 ± 20.41 *	211.45 ± 21.50 *	197.99 ± 18.51 *	210.12 ± 20.51 *
100 μM H_2_O_2_	102.43 ± 8.34	103.66 ± 9.80	103.99 ± 8.08	102.50 ± 10.50	102.50 ± 9.65
100 μM H_2_O_2_ + 3.125	127.43 ± 6.34	113.66 ± 10.48	123.99 ± 10.09	106.81 ± 10.40	131.81 ± 12.40
100 μM H_2_O_2_ + 6.25	131.32 ± 11.57 *	119.91 ± 10.50	126.02 ± 11.26	113.94 ± 11.33	163.94 ± 14.35 *
100 μM H_2_O_2_ + 12.5	144.36 ± 18.83 *	145.79 ± 12.61 *	136.81 ± 13.33 *	124.09 ± 12.64 *	234.06 ± 21.63 *
100 μM H_2_O_2_ + 25	198.44 ± 16.85 *	154.01 ± 14.94 *	188.02 ± 28.01 *	134.24 ± 24.61 *	499.24 ± 54.60 *
100 μM H_2_O_2_ + 50	319.46 ± 23.20 *	212.96 ± 19.07 *	288.29 ± 22.14 *	145.08 ± 16.06 *	766.08 ± 79.15 *
**Concentrations** **(μg/mL)**	**(B) Cell** **Apoptosis (%)**				
	**1**	**2**	**3**	**7**	**Positive Control**
**0**	2.50 ± 0.70 *	2.55 ± 0.30 *	2.40 ± 0.21 *	2.04 ± 0.24 *	2.35 ± 0.17 *
100 μM H_2_O_2_	67.53 ± 4.56	66.87 ± 3.59	64.96 ± 6.73	68.67 ± 5.98	67.23 ± 4.39
100 *μ*M H_2_O_2_ + 3.125	61.50 ± 5.73	62.50 ± 5.91	64.45 ± 3.34	66.50 ± 6.11	66.81 ± 6.41
100 *μ*M H_2_O_2_ + 6.25	34.33 ± 3.44 *	57.94 ± 5.34 *	40.32 ± 3.57 *	60.29 ± 6.07	61.94 ± 5.33
100 *μ*M H_2_O_2_ + 12.5	9.56 ± 0.99 *	46.09 ± 3.64 *	13.36 ± 2.83 *	55.13 ± 3.12 *	13.09 ± 1.63 *
100 *μ*M H_2_O_2_ + 25	2.63 ± 0.19 *	24.16 ± 2.15 *	2.44 ± 0.19 *	45.70 ± 4.13 *	3.24 ± 0.20 *
100 *μ*M H_2_O_2_ + 50	1.92 ± 0.21 *	10.68 ± 1.15 *	1.46 ± 0.20 *	23.89 ± 2.03 *	0.68 ± 0.06 *

Note: * *p* ˂ 0.05 *vs.* model group.

#### 2.7.2. DPPH Free Radical-Scavenging Activity

From the results, it was found that phenolics **1**–**3**, **7** isolated from *L. chinensis* showed obvious free radical scavenging activity on DPPH with IC_50_ values of 2.11 ± 0.18, 2.89 ± 0.28, 3.58 ± 0.18, 10.70 ± 1.18 μM, respectively. Meanwhile, phenolics **1**–**3** exhibited more potential antioxidant activity than positive control-quercetin (IC_50_: 5.40 ± 0.30 μM).

Osteoporosis is a degenerative bone disease characterized by low bone mass and structural deterioration of bone tissue, leading to bone fragility. Bone integrity requires a tight coupling between the activity of bone-forming osteoblasts and bone-resorbing osteoclasts [25]. Reactive oxygen species (ROS) are involved in osteogenesis including bone formation and resorption, which are associated with the aging process and may result in osteoporosis [26,27,28,29,30]. During bone formation, osteoblasts undergo a cascade of complex events that might include three phases: proliferation, osteogenic differentiation, and mineralization of extracellular matrix [31].

This study reported for the first time to investigate the osteogenic effects of the phenolics from the roots of *L. chinensis*. Two new phenolics were isolated from the the roots of *L. chinensis*. Anti-osteoporosis activity of the phenolics **4**–**6** was evaluated in our former research [32]. Phenolic **1** showed enhancing effects on proliferation of rat osteoblast cells at the tested concentrations (3.125–50 µg/mL). Phenolics **1**–**3** at the majority of the tested concentrations (3.125–50 µg/mL) increased the osteogenic differentiation. At 7 day after treatment, phenolic **1** at the concentration of 25 µg/mL increased the ALP activity to the highest level by 80.1%, compared to the control. Treated with 12.5 μg/mL phenolic **1**, the osteocalcin content was significantly increased by about 0.65-fold when compared with the negative control. Collagen content of the rat osteoblastic cells was determined by hydroxyproline assay. Treated with the 6.25 μg/mL phenolic **1**, the hydroxyproline content was significantly increased by about 0.44-fold when compared with the negative control. Phenolic **1** at 12.5 µg/mL increased the area of nodules by about 9.35-fold.

## 3. Experimental

### 3.1. General

Optical rotations were measured using a JASCO P-1030 (Tokyo, Japan) automatic digital polarimeter. NMR spectra (400 MHz for ^1^H NMR) were recorded on a Bruker DPX-400 spectrometer (Karlsruhe, Germany) using standard Bruker pulse programs. Chemical shifts were showed as the *δ*-value with reference to tetramethylsilane (TMS) as an internal standard. ESI-MS data were obtained on an Agilent 1200 HPLC/6410B TripleQuad mass spectrometer (Santa Clara, CA, USA), and HR-ESIMS were measured on a Bruker APEX II mass spectrometer (Bremen, Germany). Sephadex LH-20 (Pharmacia, Stockholm, Sweden), silica gel (Qingdao Ocean Chemical Co., Ltd, Qingdao, China), Octadecylsilanized (ODS) silica gel (Macherey-Nagel, Duren, Germany) were used for column chromatography. TLC was carried out on Silica gel 60 F_254_ (0.25 mm, Merck, Darmstadt, Germany), and RP-18 F_254_ (0.25 mm, Merck) plates, and spots were visualized by spraying with 15% H_2_SO_4_ followed by heating. HPLC was performed using an octadecylsilanized (ODS) silica gel column (XTerra 10 μm, 19 × 250 mm, Waters). Dulbecco’s modified Eagle’s medium (DMEM), fetal bovine serum (FBS), and trypsin-EDTA solution (1×) were obtained from Hyclone (Logan, UT, USA). Annexin-V/PI Apoptosis Detection Kit was purchased from Beyotime Institute of Biotechnology (Jiangsu, China). Hydroxyproline Assay Kit was purchased from Nanjing Jiancheng Bioengineering Institute (Nanjing, China). All other chemicals were analytical or HPLC grade and obtained from Shanghai Chemical Reagents Co., Ltd (Shanghai, China).

### 3.2. Plant Material

The fresh roots of *Livistona chinensis* (Jacq.) R. Br. were collected in Jiangmen, Guangdong Province, China, in September 2009, and were identified by Prof. Xiangjiu He, School of Pharmaceutical Sciences at Wuhan University. A voucher specimen (No. 20100115) is available at the School of Pharmaceutical Sciences, Wuhan University in Wuhan (430071), China.

### 3.3. Extraction and Isolation

The air-dried roots of *L. chinensis* (1.5 kg) were extracted with 70% EtOH (25 L × 3) at refluxed. Evaporation of the organic solvent under a vacuum at 55 °C yielded a crude extract (560 g). The concentrated brown syrup was resuspended in water and partitioned with chloroform (3 L × 3), ethyl acetate (3 L × 3) and water-saturated *n*-butanol (3 L × 3) gradually to afford 33.0 g, 35.3 g and 230.3 g of dried organic extracts, respectively. The ethyl acetate fraction with the most potential activity was fractionated over a silica gel (200 ~ 300 mesh) column eluting with a gradually amount of MeOH in CHCl_3_ to give 15 fractions. The CHCl_3_/MeOH (25:1) eluate was further purified on a silica gel column, eluted with CHCl_3_/MeOH (100:1→1:1), combined with preparative HPLC (28% methanol containing 0.1% CF_3_COOH, pH 3.0), yielding compounds **2** (56.79 mg), **5** (12.33 mg) and **7** (15.56 mg). The CHCl_3_/MeOH (10:1) eluate was subjected to an octadecylsilanized silica gel (ODS) column, followed by a preparative HPLC with 26% methanol (containing 0.1% CF_3_COOH, pH 3.0), yielding compound **6** (10.76 mg). The CHCl_3_/MeOH (15:1) eluate was subjected to silica gel and Sephadex LH-20 column chromatography, followed by preparative HPLC with 20% methanol (containing 0.1% CF_3_COOH, pH 3.0) to give phenolics **1** (15.56 mg), **3** (13.47 mg), **4** (21.34 mg) and **8** (132.46 mg).

### 3.4. (2R,3R)-3,5,6,7,3',4'-Hexahydroxyflavane (**1**)

Amorphous colorless powder; 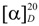 = −43.0 (c 0.3, MeOH). ESI-MS (positive-ion mode) [M+Na]^+^ *m/z* 329. ^1^H-NMR (CD_3_OD) δ_H_ 4.77 (1H, brs, H-2), 4.14 (1H, m, H-3), 2.83 (1H, dd, *J* = 16.4, 4.8 Hz, H-4ax), 2.70 (1H, dd, *J* = 16.8, 2.8 Hz, H-4eq), 5.91 (1H, s, H-8), 6.94 (1H, d, *J* =1.6 Hz, H-2'), 6.72 (1H, d, *J* = 8.4 Hz, H-5'), 6.76 (1H, dd, *J* = 8.4, 2.0 Hz, H-6'); ^13^C-NMR (CD_3_OD): δ_C_ 79.9 (C-2), 67.6 (C-3), 29.5 (C-4), 157.7(C-5), 145.8 (C-6), 146.1 (C-7), 96.6 (C-8), 158.0 (C-9), 100.3 (C-10), 132.4 (C-1'), 115.5 (C-2'), 157.4 (C-3'), 157.2 (C-4'), 116.1 (C-5'), 119.6 (C-6').

### 3.5. Anthracene-2,4,9-triol (**2**)

Amorphous colorless powder; ESI-MS (positive-ion mode) [M+Na]^+^ *m/z* 249. ^1^H-NMR (CD_3_OD) δ_H_ 6.81 (s, H-3), 6.92 (s, H-5), 6.97 (s, H-7), 6.76 (d, *J* = 8.8 Hz, H-10), 6.15 (t, *J* = 5.6 Hz, H-11), 7.34 (d, *J* = 8.4 Hz, H-12) and 6.44 (d, *J* = 2.0 Hz, H-14); ^13^C-NMR (CD_3_OD) δ_C_ 116.7 (C-1), 159.8 (C-2), 106.0 (C-3), 159.8(C-4), 130.8 (C-5), 141.6 (C-6), 102.9 (C-7), 128.9 (C-8), 158.6 (C-9), 106.0 (C-10), 128.9 (C-11), 116.7 (C-12), 127.2 (C-13), 129.4 (C-14).

### 3.6. Rat Osteoblast Cell Culture

Osteoblastic cells were enzymatically isolated from newborn rat calvaria by a previously described method with slight modifications [33]. The bone pieces were digested sequentially in a trypsin II-S (25 mg)-collagenase IA (70 mg) in 15 mL PBS solution at 37 °C for 30 min. Rat osteoblastic cells obtained from the last three digestion steps were pooled and plated together in a T25 tissue culture dish at a concentration of 50,000 cells/cm^2^ and cultured in DMEM supplemented with 10% FBS and 1% antibiotic (100 units/mL penicillin and 100 μg/mL streptomycin) at 37 °C in a 5% CO_2_ humid atmosphere. The medium was changed every 3 day to remove the non-adherent cells.

### 3.7. C2C12 Cell Culture

The C2C12 cell line was purchased from the American Type Culture Collection. Cells were cultured in basal medium, constituted with DMEM containing 10% fetal bovine serum (FBS, Gibco, Carlsbad, CA, USA) and 1% antibiotics (100 units/mL penicillin and 100 μg/mL streptomycin), for incubation at 37 °C in a 5% CO_2_ humidified atmosphere.

### 3.8. Cell Viability Assay

Cells were seeded in a 96-well plate at a density of 5 × 10^3^ cells/well. The total volume was adjusted to 100 μL with growth medium. 24 h after the seeding, the cells were exposed to the phenolics or resveratrol (positive drug) of different concentrations. At two days after treatment, cell viability was examined using a standard MTT method [34]. Drug effect was expressed as percentage relative to the controls.

### 3.9. Alkaline Phosphatase (ALP) Activity

Cells were cultured in DMEM with 10% FBS and 1% antibiotic (100 units/mL penicillin and 100 μg/mL streptomycin) at 37 °C in a humid atmosphere containing 5% CO_2_. 1 mL of 5 × 10^4^ cells/well of osteoblastic cells was seeded in 48-well plates. After 24 h, the culture medium with the phenolics or resveratrol (positive drug) at concentrations of 0, 3.125, 6.25, 12.5, 25 and 50 µg/mL was added. 

The ALP activity of the samples was determined by a colorimetric assay [35]. At 7 days after treatment, cells seeded in 48-well plates were washed twice with 50 mM PBS (pH 7.4) and kept in 0.1% Triton X-100 lysis buffer overnight at –20 °C. The cells were later thawed. 300 µL of substrate buffer (6.7 mmol/L disodium *p*-nitrophenylphosphate hexahydrate, 25 mmol/L diethanolamine and 1 mmol/L MgCl_2_) was added in. After the mixtures were incubated at 37 °C for 30 min, we measured the absorbance at 405 nm.

### 3.10. Osteocalcin Assay

Osteoblastic cells (2 mL, 1 × 10^5^ cells/well) were seeded in 6-well plates. After 24 h, the culture medium with the phenolics or resveratrol (positive drug) at 0, 3.125, 6.25, 12.5, 25, and 50 µg/mL concentrations was added in. Fourteen days after treatment, the conditioned media were collected for assessment. The concentration of the free osteocalcin was measured by radioimmunoassay (RIA) according to the manufacturer’s instructions (Tian Jin Nine Tripods Medical and Bioengineering Co, Ltd., Tianjin, China).

### 3.11. Hydroxyproline Assay

Osteoblastic cells (2 mL, 1 × 10^5^ cells/well) were seeded in 12-well plates. After 24 h, the culture medium with the phenolics or resveratrol (positive drug) at 0, 1.5625, 3.125, 6.25, 12.5, and 25 µg/mL concentrations was added. Twenty two days after treatment, cell samples were hydrolyzed in 6 N HCl (final concentration) for 12 h at 110 °C. The samples were filtered and vacuum dried. The residue was dissolved in water (200 µL). The hydroxyproline content was assayed according to the manufacturer’s instructions (Nanjing Jiancheng Bioengineering Institute, Nanjing, China).

### 3.12. Determination and Quantification of Mineralized Bone Nodules

Osteoblastic cells (2 mL, 1 × 10^5^ cells/well) were seeded in 12-well plates. After 24 h, osteogenic medium (10 mM L-glycerophosphate, 50 µg/mL ascorbic acid) with the phenolics or resveratrol (positive drug) was added. On Day 20, cells seeded in 24-well plates were fixed in 95% ethanol for 15 min and the mineralized bone nodules were visualized by alizarin red staining techniques [36]. The cells were stained with alizarin red (40 mM, pH 7.2) for 10 min and then rinsed them with PBS. Nodules were visualized using an inverted microscope. The area of mineralized nodules was quantified by the Image-Pro Plus 6.0 Software.

### 3.13. Flow Cytometric Analysis for Apoptosis

To investigate the protective effects of the new flavane on H_2_O_2_-induced cytotoxicity, C2C12 mouse myoblast cells were cultured in the presence of 100 μM H_2_O_2_ with or without treatment with the phenolics or quercetin (positive drug). Apoptosis was examined by Annexin V-fluorescein isothiocyanate staining (Beyotime Institute of Biotechnology, Jiangsu, China) according to the manufacturer’s instructions. Cells were seeded in 6-well plates. Two days after treatment, the cells were harvested by trypsinization, rinsed twice with PBS, and suspended in 500 µL of binding buffer. The suspended cells were incubated for 15 min at 4 °C with 5 µL Annexin V-FITC solution, and incubated for another 5 min at 4 °C after adding 10 µL of PI solution. The FITC fluorescence intensity of 10,000 cells was measured with a flow cytometer (Beckman-Coulter, Inc., Indianapolis, IN, USA). The apoptosis was expressed as the ratio of apoptotic cell count to total cell count.

### 3.14. DPPH Radical Scavenging Assay

The effect of the new flavane on DDPH radical was determined according to the method reported by Yen and Chen [37]. DPPH (50 mg/L) was dissolved in MeOH. The samples were dissolved in DMSO. The DPPH solution (995 μL) was mixed with 5 μL of the samples. The mixture was shaken and allowed to stand at room temperature in the dark for 20 min. The absorbance of the resulting solution was measured spectrophotometrically at 517 nm.

### 3.15. Statistical Analysis

All data were expressed as mean ± S.D. from at least three independent experiments, each performed in quintuplicate. The statistical significance among groups were performed by the SPSS 19.0 software and evaluated using variance (ANOVA) with Fisher’s PLSD test. The probabilities (P) less than 0.05 were considered statistically significant.

## 4. Conclusions

The findings of the present study showed that the phenolics in *L. chinensis* were able to activate simultaneously the rat osteoblastic cells at three phases. Phenolics **1**–**3** significantly promoted osteogenic proliferation, differentiation, and mineralization. ROS are also involved in bone resorption with a direct contribution of osteoclast-generated superoxide to bone degradation [8]. Oxidative stress is a pivotal pathogenic factor for age-related bone loss in mice, leading to an increase in osteoblast and osteocyte apoptosis and a decrease in osteoblast number and the rate of bone formation [38,39]. In addition, osteoblasts produce antioxidants such as glutathione peroxidase to protect against ROS [38,39]. The present study evaluated the antioxidative activity of phenolics through evaluating protecting activity against H_2_O_2_-induced apoptosis in C2C12 myogenic cells and DPPH free radical-scavenging activity. And it indicated that anti-osteoporosis effect of the phenolics isolated from the roots of *L. chinensis* might be linked to a reduction of oxidative stress. Further investigations are desirable to confirm this hypothesis based on findings of our current *in vitro* study.

## References

[1] Ozgocmen S., Kaya H., Fadilliogl E., Aydogan R., Yilmaz Z. (2007). Role of antioxidant systems, lipid peroxidation, and nitric oxide in postmenopausal osteoporosis. Mol. Cell. Biochem..

[2] Overton T.R., Basu T.K. (1999). Longitudinal changes in radial bone density in older men. Eur. J. Clin. Nutr..

[3] Linnane A.W., Eastwood H. (2006). Cellular redox regulation and prooxidant signaling systems, a new perspective on the free radical theory of aging. Ann. N. Y. Acad. Sci..

[4] Sendur O.F., Turan Y., Tastaban E., Serter M. (2009). Antioxidant status in patients with osteoporosis: A controlled study. Joint Bone Spine.

[5] Mody N., Parhami F., Saraflan T.A., Demer L.L. (2001). Oxidative stress modulates osteoblastic differentiation of vascular and bone cells. Free Radic. Biol. Med..

[6] Fatokun A.A., Stone T.W., Smith R.A. (2008). Responses of differentiated MC3T3-E1 osteoblast-like cells to reactive oxygen species. Eur. J. Pharm..

[7] Yang S., Madyastha P., Bingel S., Ries W., Key L. (2001). A new superoxide-generating oxidase in murine osteoclasts. J. Bio. Chem..

[8] Sontakke A.N., Tare R.S. (2002). A duality in the roles of reactive oxygen species with respect bone metabolism. Clin. Chim. Acta.

[9] Healthy Ministry of Guangzhou Force Logistics (1969). Common Chinese Herbal Medicine Handbook.

[10] Zhao G.P., Dai S., Chen E.S. (2001). Dictionary of Traditional Chinese Medicine.

[11] Chen P., Yang J.S. (2007). Studies on chemical constituents of *Livistona chinensis* seeds. Chin. Tradit. Herb Drugs.

[12] Chen P., Yang J.S. (2008). Studies on chemical constituents of *Livistona chinensis* seeds. Chin. Pharm. J..

[13] Tao Y., Yang S.P., Zhang H.Y., Liao S.G., Wei W., Yan W., Wu Y., Tang X.C., Yue J.M. (2009). Phenolic compounds with cell protective activity from the fruits of *Livistona chinensis*. J. Asian Nat. Prod. Res..

[14] Zeng X.B., Qiu Q., Jiang C.G., Jing Y.T., Qiu G.F., He X.J. (2011). Antioxidant flavanes from *Livistona chinensis*. Fitoterpia.

[15] Zeng X.B., Wang Y.H., Qiu Q., Jiang C.G., Jing Y.T., Qiu G.F., He X.J. (2012). Bioactive phenolics from the fruits of *Livistona chinensis*. Fitoterapia.

[16] Zeng X.B., Xiang L.M., Li C.Y., Wang Y.H., Qiu G.F., Zhang Z.X., He X.J. (2012). Cytotoxic ceramides and glycerides from the roots of *Livistona chinensis*. Fitoterapia.

[17] Zeng X.B., Li C.Y., Wang H., Qiu Q., Qiu G.F., He X.J. (2013). Unusual lipids and acylglucosylsterols from the roots of *Livistona chinensis*. Phytochem. Lett..

[18] Waterman P.G., Faulkner D.F. (1979). (−)-Epiafzelechin from the Root Bark of *Cassia sieberiana*. Planta Med..

[19] Jin G.Z., Jin H.S., Jin L.L. (2011). Synthesis and antiproliferative activity of 1,4-bis(dimethylamino)-9,10-anthraquinone derivatives against P388 mouse leukemic tumor cells. Arch. Pharm. Res..

[20] Aubin J.E. (1998). Advances in osteoblast lineage. Biochem. Cell. Biol..

[21] Lian B.J., Stein G.S., Canalis E., Robey P.G., Boskey A.L., Favus M.J. (1999). Bone Formation: Osteoblast Lineage Cells, Growth Factors, Matrix Proteins and the Mineralization Process. Primer on the Metabolic Bone Diseases and Disorders of Mineral Metabolism.

[22] Tong A.L., Chen L.L., Ding G.Z. (1999). Progress of mechanism of osteoblastic bone formation. Chin. J. Osteoporos..

[23] Lee N.K., Sowa H., Hinoi E., Ferron M., Ahn J.D., Confavreux C., Dacquin R., Mee P.J., McKee M.D., Jung D.Y. (2007). Endocrine regulation of energy metabolism by the skeleton. Cell.

[24] Sakano S., Murata Y., Miura T. (1993). Collagen and alkaline phosphatase gene expression during bone morphogenetic protein (BMP)-induced cartilage and bone differentiation. Clin. Orthop. Relat. Res..

[25] Riggs B.L., Melton L.J. (1992). The prevention and treatment of osteoporosis. N. Engl. J. Med..

[26] Nohl H. (1993). Involvement of free radicals in ageing: A consequence or cause of senescence. Br. Med. Bull..

[27] Basu K., Michaëlsson H., Olofsson H., Johansson S., Melhus H. (2001). Association between oxidative stress and bone mineral density. Biochem. Biophys. Res. Commun..

[28] Muthusami S., Ramachandran I., Muthusamy B., Vasudevan G., Prabhu V., Subramaniam V., Jagadeesan A., Narasimhan S. (2005). Ovariectomy induces oxidative stress and impairs bone antioxidant system in adult rats. Clin. Chim. Acta.

[29] Isomura H., Fujie K., Shibata K., Inoue N., Iizuka T., Takebe G., Takahashi K., Nishihira J., Izumi H., Sakamoto W. (2004). Bone metabolism and oxidative stress in postmenopausal rats with iron overload. Toxicology.

[30] Yalin S., Bagis S., Aksit S.C., Arslan H., Erdogan C. (2006). Effect of free radicals and antioxidants on postmenopausal osteoporosis. Asian J. Chem..

[31] Zhang D.W., Cheng Y., Wang N.L., Zhang J.C., Yang M.S., Yao X.S. (2008). Effects of total flavonoids and flavonol glycosides from Epimedium koreanum Nakai on the proliferation and differentiation of primary osteoblasts. Phytomedicine.

[32] Zeng X.B., Su Y.J., Zheng Y.Y., Cui L. (2013). Osteogenic effects of the flavanes from green tea polyphenols. Acta Pharmacol. Sin..

[33] Declercq H., Vreken N.V., Maeyer E.D., Verbeeck R., Schacht E., Ridder L.D., Cornelissen M. (2004). Isolation, proliferation and differentiation of osteoblastic cells to study cell/biomaterial interactions: Comparison of different isolation techniques and source. Biomaterials.

[34] He X.J., Liu R.H. (2007). Triterpenoids isolated from apple peels maybe responsible for their anticancer activity. J. Agric. Food Chem..

[35] Rao L.G., Liu L.J., Murray T.M., McDermott E., Zhang X. (2003). Estrogen added intermittently but not continuously, stimulates differentiation and bone formation in SaOS-2 cells. Biol. Pharm. Bull..

[36] Hale L.V., Ma Y.F., Santerre R.F. (2000). Semi-quantitative fluorescence analysis of calcein binding as a measurement of *in vitro* mineralization. Calcif. Tissue Int..

[37] Yen G.C., Chen H.Y. (1995). Antioxidant activity of various tea extracts in relation to their antimutagenicity. J. Agric. Food. Chem..

[38] Manolagas S.C. (2008). De-fense! De-fense! De-fense: Scavenging H_2_O_2_ while making cholesterol. Endocrinology.

[39] Garrett J.R., Boyce B.F., Oreffo R.O., Bonewald L., Poser J., Mundy G.R. (1990). Oxygen-derived free radicals stimulate osteoclastic bone resorption in rodent bone *in vitro* and *in vivo*. J. Clin. Invest..

